# A Blockchain-Based Federated Learning Method for Smart Healthcare

**DOI:** 10.1155/2021/4376418

**Published:** 2021-11-24

**Authors:** Yuxia Chang, Chen Fang, Wenzhuo Sun

**Affiliations:** ^1^Department of Emergency Medicine, Henan Provincial People's Hospital, Zhengzhou 450001, China; ^2^Key Laboratory of Nursing Medicine of Henan Province, Zhengzhou 450001, China; ^3^People's Hospital of Zhengzhou University, Zhengzhou 450001, China; ^4^Information Engineering University, Zhengzhou 450001, China

## Abstract

The development of artificial intelligence and worldwide epidemic events has promoted the implementation of smart healthcare while bringing issues of data privacy, malicious attack, and service quality. The Medical Internet of Things (MIoT), along with the technologies of federated learning and blockchain, has become a feasible solution for these issues. In this paper, we present a blockchain-based federated learning method for smart healthcare in which the edge nodes maintain the blockchain to resist a single point of failure and MIoT devices implement the federated learning to make full of the distributed clinical data. In particular, we design an adaptive differential privacy algorithm to protect data privacy and gradient verification-based consensus protocol to detect poisoning attacks. We compare our method with two similar methods on a real-world diabetes dataset. Promising experimental results show that our method can achieve high model accuracy in acceptable running time while also showing good performance in reducing the privacy budget consumption and resisting poisoning attacks.

## 1. Introduction

With the growth in volume and types of clinical data, there is an urgent need for efficient mining models to analyze these data so as to help disease diagnosis, provide medical solutions, and improve the medical care for patients. Machine learning is an effective tool with powerful computation capabilities, which has been used in many fields, such as image recognition, natural language processing, and healthcare. However, machine learning models can only reach high accuracy with abundant training data, which is especially important in healthcare that sometimes decides whether a patient's life can be saved. Traditional centralized training methods usually require collecting a large amount of data from a powerful cloud machine, which may cause serious user privacy leakage, especially in the medical domain. Many governments have issued laws prohibiting collecting data relevant to user privacy, such as the European Union's General Data Protection Regulation (GDPR). The emergence of the Medical Internet of Things (MIoT) empowers traditional fields such as healthcare, medical care, public health, and community services, where large numbers of MIoT devices such as wearable sensors are distributed at the edge of the network to collect patient data. Federated learning (FL) [1], as a distributed machine learning framework, can allow multiple devices to train machine learning models collaboratively without sharing their raw data, which just contributes to realizing smart healthcare in the MIoT while reducing the privacy leakage of patients.

**Figure 1:**
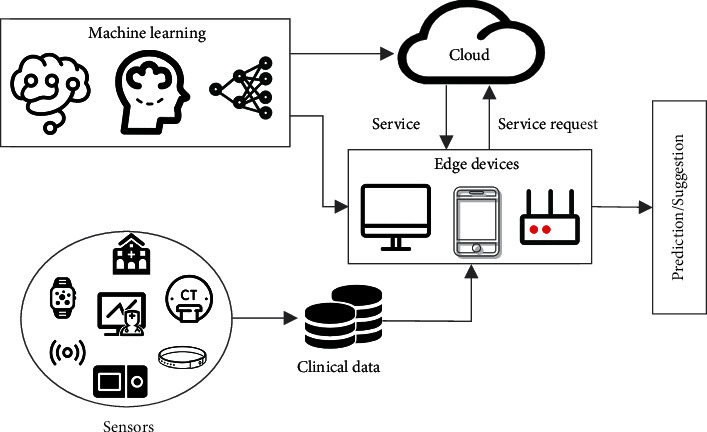
A typical FL-based smart healthcare application.

A typical FL-based smart healthcare application is shown in Figure 1, where onboard sensors collect clinical data from patients, multiple edge devices perform FL algorithm collaboratively, and the final machine learning models evaluate the patient's physical health and even request the emergency service in the cloud if necessary. However, one of the drawbacks of vanilla FL is that it needs a trustworthy central server to aggregate the model parameters uploaded by devices and distribute the global model to all devices. Once the central server is crashed by attackers, the FL training will stop. As a ledger with properties of tamper-proof, collective maintenance, and traceability, blockchain can replace the central server to decentralize the coordination process in FL, thus resisting single points of failure and illegal tampering attacks. In this way, the traditional elements in blockchain can be mapped into the training stages of FL as follows: each block represents a single training round, where the stored transactions represent model parameters uploaded by devices in that round. Then all devices can look up the model parameters from the latest block and update their local models. In view of these advantages, lots of blockchain-based FL methods have been proposed to be applied in many fields, such as smart home [2], Industrial Internet of Things (IoT) [3], and smart healthcare [4]. But with more and more advanced privacy attacks, there are still several challenges that need to be addressed while applying blockchain-based FL to healthcare: (1) the model parameters stored in the blockchain may still be stolen by attackers to infer the original private clinical data; (2) clinical data of some edge devices may be poisoned to mislead the FL process; (3) edge devices have no incentive to contribute dataset and computing power to FL.

Aimed at the above challenges, this paper integrates FL with blockchain and advanced cryptography to realize a smart healthcare model in a secure and privacy-preserving manner. The main contributions of the paper are mainly as follows:

1. We propose a blockchain-based FL framework for smart healthcare, which not only builds an accurate collaborative model based on multiple edge devices but also provides governance of the whole training process.
2. To add an extra layer of security of blockchain-based FL, we propose adaptive differential privacy (DP) algorithm that adapts noise according to the training process, balancing privacy, and model accuracy.
3. We design an efficient consensus protocol based on gradient verification, which encourages reliable MIoT devices and edge nodes to contribute their data and computing power to federated learning.

The rest of the paper is organized as follows. We introduce related works in Section 2 and give our method in Section 3.  Section 4 shows the experimental results of our method. We draw conclusions and outline future work in Section 5.

## 2. Related Work

With the development of artificial intelligence (AI), it is a common practice to deploy AI applications to assist medical diagnosis, which can improve the diagnosis rate of diseases and reduce the waiting time of patients. Dai et al. [5] turned the prediction of hospitalization task into a supervised classification problem, resulting in a considerable amount of potential saving in medical resources. Son et al. [6] developed a Support Vector Machine (SVM) model to identify predictors of medication adherence in heart failure patients. Tariq et al. [7] developed a multimodal fusion AI model from past medical data to predict the severity of COVID-19. In order to solve the problem of the absence of reliable data, Sedik et al. [8] presented a data augmentation framework to expand the limited dataset and used convolutional neural network and convolutional long short-term memory models to detect the COVID-19. However, the above-centralized training methods [5–8] need to collect sensitive clinical data in a single database, which is undesirable due to data privacy concerns. Instead, federated learning emerges as a distributed framework that performs collaborative learning while keeping all the sensitive data locally, providing a privacy-preserving solution for connecting the fragmented healthcare data on the edge devices. Many works that used FL in smart healthcare have been proposed in recent years. Qayyum et al. [9] proposed a clustered FL-based method to process clinical visual data at the edge so as to allow remote hospitals to benefit from multimodal data in a privacy-preserving manner. Brisimi et al. [10] predicted hospitalizations for patients with heart diseases by solving distributed sparse Support Vector Machine problems using FL. Xu et al. [11] gave a review for FL methods and pointed out the implications and potentials of FL in healthcare particularly. Zhang et al. [12] employed differential private generative adversarial network (DPGAN) to generate diverse patient data in a privacy-preservation way and leveraged FL to train COVID-19 models in collaboration with multiple hospitals. But these works [9–12] all need a central server to aggregate and distribute model parameters during the federated learning, which is vulnerable to a single point of failure attack.

To address this vulnerability, blockchain is introduced to enable full decentralized FL, which is also the idea of this paper. El Rifai et al. [13] integrated the FL and blockchain for the first time in a medical setting and proposed a smart contract to realize transparency and immutability while sharing knowledge. Passerat-Palmbach et al. [14] presented an advanced blockchain-orchestrated federated learning framework in medicine and outlined some challenges. Połap et al. [4] designed a multiagent medical system based on FL and blockchain that can separate complicated tasks into individual objects and process medical data in real time. While the integration of blockchain and FL can resist a single point of failure and enable life-cycle governance of the training process, due to the transparency of blockchain, it raises concerns with regard to the privacy of the model parameters. To this end, Liu et al. [15] proposed a blockchain-based secure FL framework for 5G networks, in which differential privacy noise was added on the updates to prevent inference attacks. Kumar et al. [16] proposed a blockchain-based FL framework that trained a collaborative deep learning model for COVID-19 detection using clinical data from multiple hospitals and added Laplace noise to the local gradients to ensure privacy. Rahman et al. [17] proposed a hybrid FL framework for the Internet of Health Things (IoHT) that supported lightweight DP to realize the privacy and anonymization of the IoHT data. However, differential privacy used by [15–17] will cause some loss of data utility, which will reduce the availability of smart healthcare. In this paper, we design an adaptive differential privacy algorithm to achieve a balance between data privacy and data utility.

On the other hand, poisoning attack [18] launched by malicious users is also another challenge faced by blockchain-based FL methods. Although Liu et al. [15] executed smart contracts to identify malicious participants who initiated poisoning attacks, they assumed that there was a public test dataset in advance, which was unrealistic for smart healthcare with private data of patients. In this paper, we present a simple gradient verification method that does not need a public test dataset to detect poisoning attacks.

## 3. Proposed Model

### 3.1. Threat Model

In this section, we give the threats faced by smart healthcare.

 
*Threat 1*. Potential data privacy leakage. AI models built on clinical data may be attacked by adversaries to infer patient privacy.
 
*Threat 2*. Single point of failure. Existing smart healthcare models rely on a central server to store the clinical data or exchange the model parameters. Once the central server is crashed, the model training will end with failure.
 
*Threat 3*. Poisoning attacks. Due to the vulnerability of the MIoT, adversaries may launch poisoning attacks on the MIoT device's data or local model parameters, which will compromise the correctness of FL.

For ease of understanding, the main symbols used in this paper are listed in Table 1.

### 3.2. System Architecture

**Figure 2:**
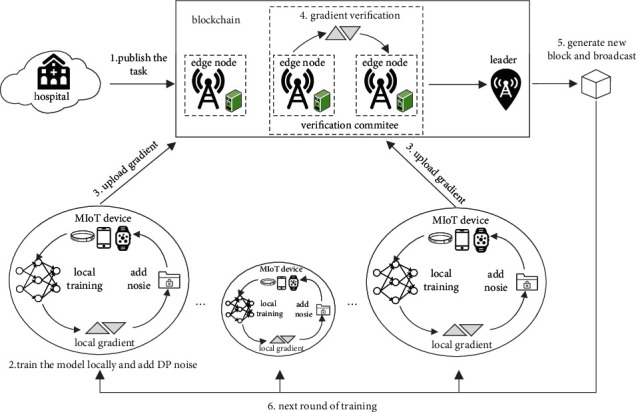
Training process in one round.

Our smart healthcare system is mainly composed of the user layer and edge node layer. The user layer mainly includes wearable sensors, MIoT devices, and mobile terminals. They are used to monitor patients' physiological condition, collect clinical data, and train FL model locally. Edge nodes are mainly composed of base stations equipped with edge computing servers that have powerful computation and communication capabilities. They maintain the blockchain as miners, receive and store the model parameters, and authenticate the parameters by consensus protocol. The training process in one round is shown in Figure 2.

As shown in Figure 2, a complete training process of one round can be formulated as the following steps:

1. Hospitals determine and send the training task to the blockchain, and then the genesis block is created and distributed to all the MIoT devices and edge nodes to perform model initialization. The genesis block mainly contains the following information: ① initial model parameters *w*_0_ and total training rounds *T*; ② public keys of all parties that are used to create signatures; ③ initial reputation value of all edge nodes and MIoT devices; ④ reputation update function.
2. The MIoT device trains the model locally based on its collected clinical data and adds DP noise to the local gradient (see details in Section 3.3.1) so as to cope with Threat 1.
3. The MIoT device uploads the noised gradient along with the signature to its associated edge node in the form of transactions.
4. After receiving data from devices within their coverage, the edge nodes first verify the legality of the signature and then elect a verification committee to detect whether the local gradients are poisoned update (see details in Section 3.3.2) so as to cope with Threat 3.
5. A leader is randomly selected to generate a new block containing the necessary model parameters for this training round. The verification committee verifies the new block and broadcasts the valid one to synchronize the ledgers of all edge nodes (see details in Section 3.3.2) so as to cope with Threat 2.
6. The MIoT device downloads the latest block from its associated edge node and updates its local model by the global gradient stored in the block. The next training round starts from step 2 until the model converges or the maximum rounds are reached.

Next, we will introduce the main construction of our method in detail.

### 3.3. Construction of Method

#### 3.3.1. Adaptive Differential Privacy Algorithm

The advanced privacy attacks such as model inversion [19] and model extraction attack [20] have shown that the model parameters stored in the blockchain are not enough to protect the privacy of raw clinical data. References [18, 21] used Shamir secret sharing and threshold Paillier encryption to protect local gradients, respectively, but both consume large computation overhead. In contrast, differential privacy technology needs less computation overhead, which is more suitable for MIoT devices with limited resources.

In principle, DP is a strictly provable mathematical framework whose basic idea is to add carefully designed noise to the input or output of a function so that the modification of any individual sample in the dataset will not have a significant impact on the output. The related definitions are as follows.

**Definition 1 .**

(differential privacy [22]). A randomized algorithm *A* : *D*⟶*R* is (*ε*, *δ*)-differentially private if for any two datasets *D* and *D*′ differing in an individual sample and any output *O* ∈ *R*:

(1) $$\begin{array}{c} \mathrm{Pr}\left[A\left(D\right)=O\right]\leq e^{\varepsilon }\times \mathrm{Pr}\left[A\left(D^{\prime }\right)=O\right]+\delta , \end{array}$$

where *ε* is the privacy budget. A small *ε* means a higher level of privacy preservation but greater accuracy loss for algorithm *A* and vice versa. *δ* is the probability that measures the violation of the “pure” differential privacy, which is usually a small value.

**Definition 2 .**

(sensitivity [22]). For any real-valued function *f* : *D*⟶*R*^*d*^ with *D* as the input dataset and *R*^*d*^ as the *d*-dimensional vector output, the sensitivity of *f* is

(2) $$\begin{array}{c} \Delta f=\underset{D,D^{\prime }}{\max}{\left\|f\left(D\right)-f\left(D^{\prime }\right)\right\|}_{p}, \end{array}$$

where *D* and *D*′ are two adjacent datasets differing in an individual sample and ‖•‖_*p*_ denotes the *L*_*p*_ norm.

**Definition 3 .**

(Gaussian mechanism [22]). Assume that *L*_2_ norm is used to compute the sensitivity of function *f*. (*ε*, *δ*)-differentially privacy can be realized via adding Gaussian noise to the output of function *f*:

(3) $$\begin{array}{c} A\left(D\right)=f\left(D\right)+N\left(0,{\left(\Delta f\sigma \right)}^{2}I\right), \end{array}$$

where *N*(0, (Δ*fσ*)^2^*I*) is the Gaussian distribution with mean 0 and standard deviation Δ*fσ* and *I* is the identity matrix.

From the above definitions, we can see that the private information in a dataset can be hidden by adding noise, but at the same time, the noise will lower the data utility. Reference [23] added noise on the raw data by local differential privacy, but it reduced the model accuracy severely. Reference [24] added Gaussian noise on the clipped gradient but did not explain how to select the clipping threshold. The value of the threshold is important to the FL model: too large a value will add excessive noise and too small a value will over clip the gradient, both of which will cause serious accuracy loss. Aimed at this issue, we draw on the idea of the RMSProp optimization algorithm and propose an adaptive differential privacy algorithm for MIoT devices, which can flexibly adjust the clipping threshold according to the training process to reduce the negative impact of noise on the model accuracy.

RMSProp is a variant of gradient descent algorithm for machine learning, which speeds up the convergence rate by adjusting the step size. The iteration formula is as follows:

(4) $$\begin{array}{c} E{\left[g^{2}\right]}_{t}\leftarrow \left(1-\gamma \right)E{\left[g^{2}\right]}_{t-1}+\gamma {\left(g_{t}\right)}^{2},\\ \theta_{t}\leftarrow \theta_{t-1}-\eta \frac{g_{t}}{\sqrt{E{\left[g^{2}\right]}_{t}+\varepsilon_{0}}}, \end{array}$$

where *θ*_*t*_ is the model parameter in the *t*-th iteration, *g*_*t*_ is the gradient, *η* is the learning rate, *𝔼*[*g*^2^]_*t*−1_ is the cumulative square of the historical gradient, *γ* is an exponent of gradient accumulation, and *ε*_0_ is to ensure that the divisor is not zero, generally set to 10^−8^. Due to the continuity and gradualness of the convergence process [25], the historical gradient can usually be used to estimate the current gradient. Therefore, *𝔼*[*g*^2^]_*t*−1_ in the RMSProp algorithm can be regarded as the prior knowledge of the current gradient.

The existing method [26] lets $C\approx {\left\|{\tilde{g}}_{t}\right\|}_{2}$ be the approximate optimal value of the clipping threshold. But according to the training process in Figure 2, the MIoT device cannot obtain the global gradient of the current training round before uploading the local gradient. So based on the idea of RMSProp, this paper uses the prior knowledge $𝔼{\left[{\tilde{g}}^{2}\right]}_{t-1}$ to predict the global gradient ${\tilde{g}}_{t}$ of the current round and then sets ${\tilde{g}}_{t}$ as the clipping threshold; that is, $C_{t}=\beta \sqrt{𝔼{\left[{\tilde{g}}^{2}\right]}_{t-1}}$, where *β* denotes the local clipping factor, and the prior knowledge $𝔼{\left[{\tilde{g}}^{2}\right]}_{t-1}$ is computed as follows:

(5) $$\begin{array}{c} E{\left[{\tilde{g}}^{2}\right]}_{0}=\overset{⟶}{0},\\ E{\left[{\tilde{g}}^{2}\right]}_{t-1}\leftarrow \left(1-\gamma \right)E{\left[{\tilde{g}}^{2}\right]}_{t-2}+\gamma {\left({\tilde{g}}_{t-1}\right)}^{2}. \end{array}$$

Note that the prior knowledge $𝔼{\left[{\tilde{g}}^{2}\right]}_{0}=0$ in the first training round will result in $C_{1}=\beta \sqrt{𝔼{\left[{\tilde{g}}^{2}\right]}_{0}}=0$, which cannot be used for gradient clipping. Therefore, we set another prior threshold *G*: when the prior knowledge of the gradient is insufficient in the initial training stage (i.e., $𝔼{\left[{\tilde{g}}^{2}\right]}_{t-1}<G$), set the gradient clipping threshold as a fixed value *C*; when the training continues until the prior knowledge satisfies $𝔼{\left[{\tilde{g}}^{2}\right]}_{t-1}>G$, set the gradient clipping threshold as $C_{t}=\beta \sqrt{𝔼{\left[{\tilde{g}}^{2}\right]}_{t-1}}$. *G* usually takes an empirical value according to the training process of the model, which may vary in different datasets, but a simple way is to set *G* as the prior knowledge $𝔼{\left[{\tilde{g}}^{2}\right]}_{t-1}$ in a certain training round. So we have

(6) $$\begin{array}{c} C_{t}=\left\{\begin{array}{cc} C, & \text{when }E{\left[{\tilde{g}}^{2}\right]}_{t-1}<G,\\ \beta \sqrt{E{\left[{\tilde{g}}^{2}\right]}_{t-1}}, & \text{when }E{\left[{\tilde{g}}^{2}\right]}_{t-1}>G. \end{array}\right) \end{array}$$

Then, in the *t*-th training round, the MIoT device *i*(1 ≤ *i* ≤ *K*) clips the local gradient *g*_*i*,*t*_ and adds DP noise as follows:

(7) $$\begin{array}{c} {\bar{g}}_{i,t}=\frac{g_{i,t}}{\max\left\{1,{\left\|g_{i,t}\right\|}_{2}/C_{t}\right\}}+N\left(0,C_{t}^{2}\sigma^{2}\right). \end{array}$$

Since the value of $\sqrt{𝔼{\left[{\tilde{g}}^{2}\right]}_{t-1}}$ in equation (6) decreases as the model converges, the local clipping threshold *C*_*t*_ will also decrease, making the DP noise *ξ* ~ *N*(0, (*C*_*t*_*σ*)^2^*I*) in equation (7) less, which contributes to the convergence of the model in the later training stage.

#### 3.3.2. Consensus Protocol Based on Gradient Verification

Since MIoT devices are widely distributed in the open network edge, the clinical data they collect may be of low quality and even be poisoned by adversaries, and then the local gradient trained on this kind of data will deviate from the global convergence trend. To remedy the adverse effects of these malicious gradients on the blockchain-based FL, we integrate gradient verification with consensus protocol to carry out a consensus process among the edge nodes. Each edge node identifies and removes malicious gradients uploaded by its associated MIoT devices so as to only aggregate qualified gradients to generate the global model and achieve reliable FL. Unlike the proof-of-work (PoW) protocol which consumes a lot of computing resources, our protocol is improved on Algorand [27]. In each round of training, only some miners are selected to verify the new block by Byzantine agreement protocol, and the communication overhead among miners is further reduced, so the consensus efficiency is high and the forking probability is extremely low. The specific details are as follows:

*(1) Initialization*. A group of edge nodes with powerful computation and communication capabilities are chosen as miners. These miners not only generate or verify block but also execute gradient verification. To ensure the security of the blockchain, we assume that, at any point, no more than 1/3 of the miners are malicious. In addition, we assign an initial reputation value to each miner. If a miner is identified by other miners to return a falsified verification result or a fake block, then its reputation value will decrease by 1.

**Figure 3:**
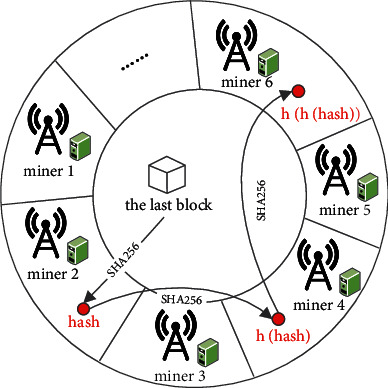
The selection of the verification committee.

*(2) Gradient Verification*. After receiving the data from its associated MIoT devices, the miner first verifies the legality of the sender by checking the digital signature. If the signature is valid, then the miner puts the local gradient into the transaction pool. Subsequently, some miners are selected to form a verification committee, which is responsible for identifying and filtering malicious gradients. In this paper, we present a reputation-based consistent hashing protocol to designate the verifier role to some miners. Specifically, given a hash ring whose space is assigned to miners in proportion to their reputation value, we repeatedly rehash the initial SHA-256 hash of the last block and map the result to the hash ring. The miner corresponding to the space where the hash lies is chosen to be the member of the verification committee. This step is repeated until the size of the committee *M* is reached, which is shown in Figure 3. The principle of the above process is similar to that of Algorand [27]: the probability of a party being selected is proportional to its reputation. Since the adversary cannot obtain the state of the block until it is generated, they cannot predict the output of the consistent hashing and launch targeted attacks.

The verification committee executes the multi-KRUM algorithm [28] on gradients in the transaction pool and accepts the top majority of the gradients in each training round. The specific process is as follows:

*Step 1*. Assume that *R* is the total number of gradients in the transaction pool and *f* is the number of Byzantine gradients. The verifier adds up the Euclidean distance of each gradient to its closest *R*-*f*-2 gradients and uses the sum as the quality score of the gradient:
  (8) $$\begin{array}{c} s\left(i,t\right)=\sum_{i⟶j}\left\|{\bar{g}}_{i,t}-{\bar{g}}_{j,t}\right\|. \end{array}$$
*Step 2*. The verifier selects the *R*-*f* gradients with the lowest scores as qualified gradients and signs them using its public key. To prevent some malicious verifier from arbitrarily accepting the gradients from its colluding MIoT devices, we require that an MIoT device's gradient must be signed by most verifiers before it is finally accepted.

**Figure 4:**
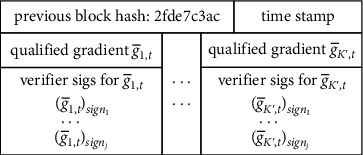
Block contents at round *t*.

*(3) Candidate Block Verification*. A miner is randomly chosen from the verification committee as the leader of the current training round. The leader collects qualified gradients in the transaction pool and generates a new block shown in Figure 4, from which it can be seen that, except for the hash value used to link the previous block, the block also contains all the qualified gradients and corresponding signatures of the verification committee. Then the new block along with the signature of the leader is sent to the verification committee to verify the validity of the block, mainly by checking the signature of the leader and verifiers. Only when more than 2/3 of the verifiers agree on the block, the block is determined to be valid and broadcasted to arrive at a consensus in the blockchain through the popular gossip protocol [29]. Otherwise, an empty block is created.

*(4) Global Model Training*. All the MIoT devices download the latest block from the blockchain, compute the global gradient by averaging all the qualified gradients stored in the block, and then update their local models. The next training round will begin until the model converges or reaches the maximum number of rounds. Note that, in each round of training, the reputation value of the MIoT device whose local gradient is identified as qualified and the verifier who returns the correct verification result will both increase by 1; otherwise, their reputation value will decrease by 1. When the reputation value decreases to zero, the entity (e.g., MIoT device or edge node) is put into the blacklist and prohibited from participating in the consensus.

The security of the above consensus protocol can be guaranteed from the following aspects: (1) In each round of training, we use consistent hashing to select different miners to verify the new block. The output of consistent hashing cannot be predicted by attackers in advance, so attackers cannot launch targeted attacks on specific verifiers. In addition, as designed by the consensus protocol, the probability of a miner being selected as a verifier is proportional to its reputation value, so attackers cannot increase the probability of being selected through Sybil attack without increasing its own reputation value, which further strengthens the security. (2) We require that an MIoT device's gradient can be identified as qualified only when it owns the signatures of most verifiers so as to prevent some malicious verifiers from colluding with some MIoT devices. (3) The consensus protocol follows Algorand [27], requiring that the newly generated block can only be identified as valid and broadcasted after it is approved by more than 2/3 of the verifiers, so its security is equivalent to that of Algorand.

### 3.4. Security Analysis

Our scheme uses a differential privacy mechanism to protect data privacy, so how to track the accumulated privacy loss during training under a given privacy budget is very important. In this paper, we use the privacy accountant proposed by Abadi et al. [24] to compute the privacy loss, which is used by many related works [15, 17]. Related definitions are as follows.

**Definition 4 .**

(privacy loss). Assume that *A* : *D*⟶*R* is a randomized algorithm, *D* and *D*′ are adjacent datasets differing in an individual sample, and then the privacy loss of output *O* ∈ *R* is

(9) $$\begin{array}{c} c\left(o,A,D,D^{\prime }\right)≜\;\log\frac{\mathrm{Pr}\left[A\left(D\right)=o\right]}{\mathrm{Pr}\left[A\left(D^{\prime }\right)=o\right]}. \end{array}$$

**Definition 5 .**

(moment accountant). The moment accountant of algorithm *A* at the *λ*th moment is defined as

(10) $$\begin{array}{c} \alpha \left(\lambda \right)≜\underset{D,D^{\prime }}{\max}\log\;E_{O∼A\left(D\right)}\left[\exp\left(\lambda c\left(O,A,D,D^{\prime }\right)\right)\right]. \end{array}$$

**Theorem 1 (composability)..**

Assume that algorithm *A* is composed of a sequence of subalgorithms *A*1, *A*2,…, *Ak*. For any moment *λ*, the moment accountant of *A* is bounded by the sum of moment accountant of *A1*, *A*2,…, *Ak*:

(11) $$\begin{array}{c} \alpha_{A}\left(\lambda \right)\leq \sum_{i=1}^{k}\alpha_{A_{i}}\left(\lambda \right). \end{array}$$

**Theorem 2 (tail bound)..**

For any *ε* > 0, the algorithm *A* is (*ε*, *δ*)-differentially private for

(12) $$\begin{array}{c} \delta =\underset{\lambda }{\min}\exp\left(\alpha_{A}\left(\lambda \right)-\lambda \varepsilon \right). \end{array}$$

According to Theorem 1, the privacy loss of our method is proportional to the number of MIoT devices and training rounds. Assume that the number of MIoT devices is *K* and training rounds is *T*. Let the overall moment accountant be *α*(*λ*) and the moment accountant of device *i*(1 ≤ *i* ≤ *K*) in the *t*-th round be *α*_*i*,*t*_(*λ*). Based on Theorem 1, we have

(13) $$\begin{array}{c} \alpha \left(\lambda \right)\leq \sum_{t=1}^{T}\sum_{i=1}^{K}\alpha_{i,t}\left(\lambda \right), \end{array}$$

where *α*_*i*,*t*_(*λ*) mainly keeps track of the DP noise *ξ* ~ *N*(0, (*C*_*t*_*σ*)^2^*I*) added on the clipped gradient of devices, shown as equation (7). The computation of *α*_*i*,*t*_(*λ*) is as follows.

Let *μ*_0_ and *μ*_1_ be the probability density function of Gaussian distribution *N*(0, (*C*_*t*_*σ*)^2^) and *N*(1, (*C*_*t*_*σ*)^2^), respectively. *μ* denotes the mixed Gaussian distribution *μ*=(1 − *q*)*μ*_0_+*qμ*_1_ of *μ*_0_ and *μ*_1_, where *q* is the sampling probability of local training. Then we need to compute *α*_*i*,*t*_(*λ*)=log  max(*E*_1_, *E*_2_), where

(14) $$\begin{array}{c} E_{1}=E_{x∼\mu_{0}}\left[{\left(\frac{\mu_{0}\left(x\right)}{\mu \left(x\right)}\right)}^{\lambda }\right], \end{array}$$

(15) $$\begin{array}{c} E_{2}=E_{x∼\mu }\left[{\left(\frac{\mu \left(x\right)}{\mu_{0}\left(x\right)}\right)}^{\lambda }\right]. \end{array}$$

Since the noise distribution *ξ* ~ *N*(0, (*C*_*t*_*σ*)^2^*I*) added on the local gradient is the same for all MIoT devices, the computation of *α*_*i*,*t*_(*λ*), 1 ≤ *i* ≤ *K*, 1 ≤ *t* ≤ *T* is the same for all devices. By equation 13, it suffices to compute or bound the overall moments *α*(*λ*) of our method. Then we can use the tail bound in Theorem 2 to convert the moment bounds to (*ε*, *δ*=min_*λ*_exp(*α*(*λ*) − *λε*))- differential privacy guarantee. Note that, in the execution of DP-based deep learning methods [24], the value range of integer *λ* is usually 0 ≤ *λ* ≤ 100.

## 4. Experiments

We want to demonstrate the following points when designing the evaluation of our method: (1) Our method can make a tradeoff between the model accuracy and privacy preservation. (2) Given a reasonable privacy budget, the running time of our method is less than similar blockchain-based FL methods. (3) Our method is robust to poisoning attack.

1. Models and datasets: the experiments are conducted under Ubuntu 18.04 system with Intel i7-8700K CPU, GTX 1080T GPU, and 16 GB RAM. We implement a small blockchain prototype based on Ethereum in Go language and train the deep learning model in Python. *go-python v1.0* [30] library is used to interface between Python and Go. We use a convolutional neural network (CNN) composed of two 5 × 5 convolution layers, a full-connected layer and a softmax output layer (1,663,370 parameters), as the deep learning model, in which the model weights are initialized by normal distribution *N*(0,0.022) and the biases are initialized as 0. As for the experimental dataset, a diabetes dataset from the American National Institute of Diabetes and Digestive and Kidney Diseases available online [31] is employed, which is composed of eight medical predictor variables and a target variable shown in Table 2, aiming to predict whether the patient has diabetes. We split the dataset into training and testing datasets in the ratio of 70 : 30. In order to simulate 20 distributed MIoT devices in smart healthcare, we randomly shuffle and divide the dataset into 20 parts evenly, and each part is regarded as the local clinical data of an MIoT device.
2. Hyperparameters and baselines: each MIoT device trains the model locally with the batch size of 64 and local iterations of 20, and the gradient parameters are transformed into byte streams for transmission by pickle module. The hyperparameters in the adaptive DP algorithm are set as follows: *G*=10^−6^, *β*=1.2, *σ*=4, *δ*=10^−4^, *γ*=0.1, *C*=3. Unless stated otherwise, we set the privacy budget *ε*=3 as default. In order to provide a comparison for our method, we choose two methods as the baseline: (1) BlockFL [32]: a blockchain-based FL method running on a device; (2) original FL [1]: the original federated learning method without any additional privacy-preserving strategies.

### 4.1. Model Accuracy

**Figure 5:**
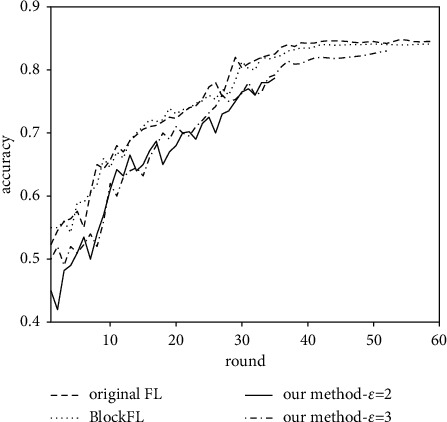
Accuracy of the three methods.

Given two different privacy budgets, we compare the model accuracy of our method with BlockFL and original FL, as shown in Figure 5.

We can find the following:

1. Our method exhausts the privacy budget *ε*=2 and *ε*=3 in the 36th and 53rd rounds and achieves model accuracy of 78.5% and 82.7%, respectively. It can be seen that the larger the privacy budget, the higher the model accuracy but the lower the level of privacy preservation simultaneously. In order to balance the model accuracy and data privacy, we set the privacy budget *ε*=3 in the rest of the experiments unless stated otherwise.
2. Original FL and BlockFL achieve higher model accuracy than our method; this is because our method adds DP noise on the gradient while the other two methods preserve the raw gradient. But given an appropriate privacy budget, our method protects the data privacy with only a slight accuracy loss. For example, when the privacy budget *ε*=3, our method achieves 82.7% accuracy, which is only slightly lower than 84.5% of original FL and 84% of BlockFL.

### 4.2. Running Time

**Figure 6:**
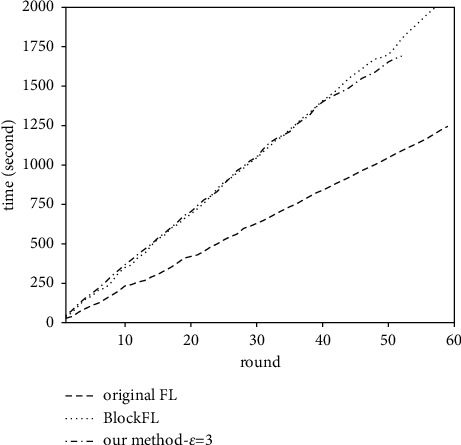
Running time of the three methods.

In order to evaluate the introduction of blockchain on the training efficiency of federated learning, we compare the running time of the three methods, as shown in Figure 6, from which we can see that the running time of BlockFL and our method is greater than that of original FL. For example, when the training reaches 50 rounds, the running time of original FL, BlockFL, and our method is 1047 s, 1702 s, and 1624 s, respectively. This is because the consensus protocol in the blockchain involves time-consuming operations such as block generation, verification, and broadcast. Therefore, blockchain-based FL methods (i.e., BlockFL and our method) achieve a series of security attributes such as auditability, reliability, and resistance to a single point of failure at the cost of some computation overhead, so they are more suitable for fields with high-security requirements, such as the medical field.

**Figure 7:**
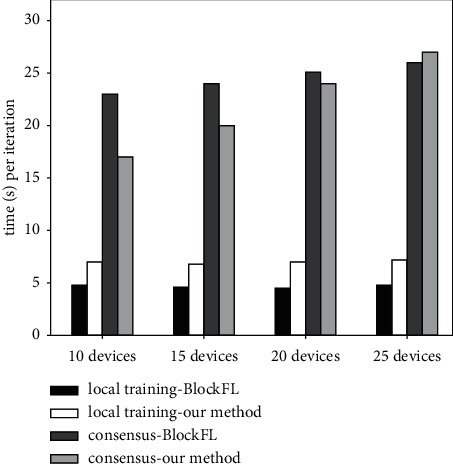
Time comparison of each training stage of BlockFL and our method.

Since blockchain-based FL methods usually include local training phase and consensus phase, Figure 7 compares the average running time per round of each phase for BlockFL and our method under different number of local devices. It can be seen that, due to the designed adaptive DP algorithm, the local training phase of our method consumes slightly more time than that of BlockFL, which does not have any additional privacy-preserving mechanism, but their local training time does not increase with the number of devices. On the contrary, their consensus time is proportional to the number of devices, and the consensus time of our method is less than that of BlockFL. This is because the PoS consensus protocol used by BlockFL needs to continuously compute nonce until reaching the target condition, which is time-consuming, while our method uses a more efficient consistent hashing protocol and gradient verification method.

### 4.3. Privacy Budget Consumption

**Figure 8:**
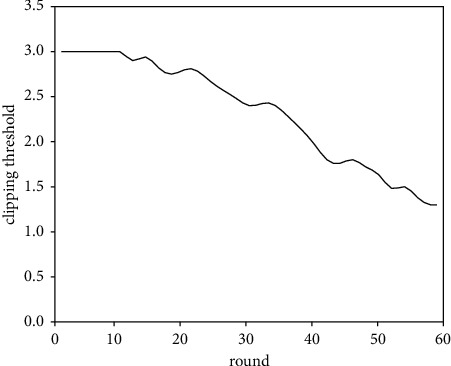
The change of clipping threshold during the training.

In the designed adaptive differential privacy algorithm, we adjust the clipping threshold *C*_*t*_ according to the training process.  Figure 8 shows the change of *C*_*t*_ during the training. We can find that, in the first 10 rounds, *C*_*t*_ keeps unchanged, which is because we fix it as 3 in the initial training stage according to equation (6). As the training goes on, the value of *C*_*t*_ gradually decreases.

In order to further measure the effect of the designed adaptive differential privacy algorithm in reducing privacy budget consumption, we compare our method with the conventional DP-based method, which fixes the clipping threshold as *C* = 3. We record the privacy budget consumed by the two methods when reaching the specified model accuracy, as shown in Table 3, where *ε*_*D*_ and *ε*_*A*_ denotes the privacy budget consumed by the conventional method and our method, respectively.

From Table 3, we can see that our method consumes much less privacy budget than the conventional method to reach the same model accuracy. For example, when the model accuracy is 80%, 82%, and 84%, our method reduces the privacy budget by 57%, 96%, and 81%, respectively, compared with the conventional method.

**Figure 9:**
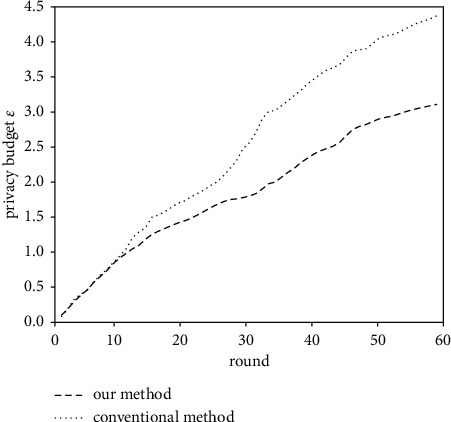
Privacy budget consumption of our method and conventional method.

Figure 9 further shows how the privacy budget of the two methods consumes during the training process. It can be seen that the curves of the two methods almost overlap at the beginning, but the increase of the privacy budget of our method gradually decreases in the later stage of training, while the privacy budget of the conventional method still increases linearly. This proves that our method uses the same fixed clipping threshold as the conventional method due to insufficient prior knowledge at the beginning but adopts the adaptive DP algorithm in the latter to reduce the consumption of the privacy budget.

### 4.4. Resistance to Poisoning Attack

Since MIoT devices are usually located at the edge of an open network, they may face poisoning attacks from adversaries. In order to evaluate the ability of our method to resist poisoning attacks, we use a label flipping attack to generate poisoned samples by changing the labels of training samples and keeping the sample features unchanged. Then, we assign the poisoned samples to the designated MIoT devices and define the attack success rate as the proportion of incorrectly predicted samples on the test dataset. We set the proportion of poisoned MIoT devices as 30% and took an average of 20 experiments as the final results.

**Figure 10:**
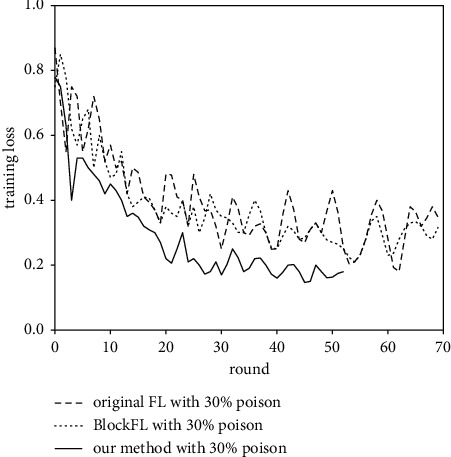
The training process of three methods under 30% poisoning attack.

Figure 10 shows the training loss of the three methods, from which we can see that, due to the limit of privacy budget *ε*=3, our method converges in 53 rounds, but the other two methods cannot converge even within 70 rounds.

**Figure 11:**
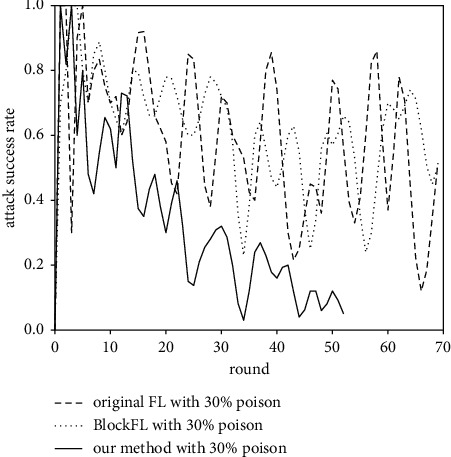
Attack success rate against three methods.

Figure 11 further shows the attack success rate of the label flipping attack on the three methods. Since the original FL and BlockFL lack a mechanism to detect the poisoned gradient, their attack success rate is almost always greater than 50%. However, our method limits the attack success rate to less than 20% in the later stage of training, and experimental data shows that four MIoT devices have been put into the blacklist at the end of the training, indicating that the consensus protocol based on gradient verification we design can effectively resist a certain proportion of poisoning attack.

## 5. Conclusions

In order to make full use of clinical data to improve the accuracy of disease diagnosis and medical service, smart healthcare based on MIoT has been widely exploited in recent years. However, it still faces challenges such as patient privacy leakage and various attacks from adversaries. To this end, we propose a blockchain-based federated learning method for smart healthcare. In particular, we design an adaptive differential privacy algorithm to carefully adjust the amount of noise added on the gradient to strike a balance between the privacy budget and accuracy degradation. The FL process is managed by a verification-based consensus protocol to prevent poisoning attacks and single point of failure. The experimental results on a real-world diabetes dataset show that our method can achieve similar accuracy to the original FL in acceptable running time. We also illustrate its ability to reduce the privacy budget consumption and withstand poisoning attacks. In the future, we will continue to explore and advance our method with public partners to make more improvements in smart healthcare.

## Figures and Tables

**Table 1:** Main symbols.

Symbols	Definitions
*T*	The total number of training rounds
*K*	The number of MIoT devices
*M*	The size of the verification committee
*g*_*i*,*t*_	The local gradient of *i*-th device in the *t*-th training round
${\bar{g}}_{i,t}$	The noised gradient of *i*-th device in the *t*-th training round
*C*_*t*_	The gradient clipping threshold in the *t*-th training round
*G*	Prior threshold
*ε*	Privacy budget
*δ*	Violation probability of the “pure” differential privacy

**Table 2:** Experimental dataset.

November	Field name	Data type
1	Pregnanci	Integer
2	Glucose	Integer
3	BloodPressure	Integer
4	SkinThickness	Integer
5	Insulin	Integer
6	BMI	Integer
7	DiabetesPedigreeFunction	Integer
8	Age	Integer
9	Outcome	Integer

**Table 3:** Privacy budget consumed by our method and conventional method.

Accuracy	*δ*	*ε*_*D*_	*ε*_*A*_
0.75	10^–4^	1.78	1.28
0.78		2.40	1.95
0.80		3.89	2.48
0.82		5.71	2.92
0.84		7.64	4.21

## Data Availability

The diabetes dataset is publicly available at https://www.kaggle.com/uciml/pima-indians-diabetes-database. Other data in this paper come from the data statistics of the test process. All the data are real and can be used.
